# Tracking the Brain State Transition Process of Dynamic Function Connectivity Based on Resting State fMRI

**DOI:** 10.1155/2019/9027803

**Published:** 2019-10-07

**Authors:** Chang Liu, Jie Xue, Xu Cheng, Weiwei Zhan, Xin Xiong, Bin Wang

**Affiliations:** ^1^Faculty of Information Engineering & Automation, Kunming University of Science and Technology, Kunming, China; ^2^College of Information and Network Security, Yunnan Police College, Kunming, China; ^3^National Engineering Laboratory for Big Data Application Technology for Improving Government Governance Capability, Guiyang, China

## Abstract

BOLD-fMRI technology provides a good foundation for the research of human brain dynamic functional connectivity and brain state analysis. However, due to the complexity of brain function connectivity and the high dimensionality expression of brain dynamic attributions, more research studies are focusing on tracking the time-varying characteristics through the transition between different brain states. The transition process is considered to occur instantaneously at some special time point in the above research studies, whereas our work found the brain state transition may be completed in a time section gradually rather than instantaneously. In this paper, a brain state conversion rate model is constructed to observe the procedure of brain state transition trend at each time point, and the state change can be observed by the values of conversion rate. According to the results, the transition of status always lasts for a few time points, and a brain state network model with both steady state and transition state is presented. Network topological overlap coefficient is built to analyze the features of time-varying networks. With this method, some common regular patterns of time-varying characteristics can be observed strongly in healthy children but not in the autism children. This distinct can help us to distinguish children with autism from healthy children.

## 1. Introduction

Autism spectrum disorder (ASD) is considered as a relatively serious developmental disorder in brain [1], usually congenital and found in early childhood development [2, 3]. It is currently one of the most severe childhood psychiatric diseases, which mainly manifested in social and communication dysfunction [4], language dysfunction [5], and repetitive ritualized stereotypes [6]. As the prevalence of ASD increased in recent years [7], more and more researchers devoted themselves to explore the etiology of ASD [8–10].

As a popular imaging technique, resting-state functional magnetic resonance imaging (rs-fMRI) is applied to discover human brain functional organization characters. It is also used to distinguish brain diseases [11–15]. The data-driven methods were used to build large-scale brain functional networks, and then the hierarchy clustering was adopted to find the differences in network connectivity patterns between ASD patients and healthy controls (HC) [16]. By using independent component analysis (ICA)for default mode network (DMN) functional connectivity in children brain based on rs-fMRI, some changes were found between normal developed children and those with ASD [17, 18]. An analysis of the intrinsic connectivity networks (ICNs) both in ASD and in HC was carried out; furthermore, research on functional connectivity and spatial overlap between these common ICNs revealed the distinction in omics connectivity characteristics between ASD patients and HC [19, 20]. Local and long-distance connections during the process of brain activity were measured by using a common graph theory framework, and those results were used to examine the spontaneous characteristics of brain activity between ASD and HC [21]. An enhanced effect size threshold (EEST) method was used for extracting connectivity-based features based on rs-fMRI in order to diagnose ASD automatically [22].

Many fMRI studies focused on blood oxygenation level dependent (BOLD) signals indicate that brain activities change as time lapses [23–25] and functional connectivity networks built on BOLD-fMRI signals can also reflect time-varying characteristics [26–28]. Sliding window technology is used in some studies to perform dynamic information analysis on BOLD signals which acquisited from rs-fMRI data [29–31]. And then some correlation analysis methods are adopted to construct dynamic brain function network such as Pearson correlation analysis [32], nonlinear Spearman rank correlation analysis [33], and partial correlation analysis [34]. In order to study the time variability of human brain function network and further identify brain diseases, some studies have tried to express the characteristics of brain state of dynamic brain function connectivity networks. The hidden Markov model (HMM) is used to estimate the states of brain in different time on the basis of BOLD-fMRI signal analysis [28]. Some modularization and topological clustering methods are applied for dynamic brain function connectivity to find the status distinct over time [35]. K-means algorithm also is widely used to distinguish one brain status from another.

The existing research studies the changes in brain function network over time by constructing a dynamic brain function network, identifying brain state [36–38], and effectively identifying brain diseases. However, in the research of clustering and recognizing brain state, the dimension of brain function network is considered too high, and it is difficult to directly observe its main features, which brings difficulties to further study the dynamic characteristics of brain network. The network characteristics of state transitions are rarely considered in the subsequent state recognition process.

This paper analyzes the dynamic characteristics of brain network state by performing dimensionality reduction clustering on high-dimensional dynamic brain networks, it analyzes the state transition critical point statistically and establishes the instantaneous conversion rate model to observe the transition trend of dynamic brain network state. Based on this, this paper proposes a conjecture that the brain network state transitions in a range, but not instantaneously, constructs a brain state network and uses the network topology coefficients to calculate the network similarity in the brain state network to express the time-varying characteristics of the brain state network. The method was used to analyze the difference in the time-varying characteristics of brain state network between healthy children and children with autism, and the effectiveness of the proposed method was verified.

## 2. Materials and Preprocessing

### 2.1. Participants

The rs-fMRI dataset used in this study is obtained from the ABIDE database [39]. A total of 40 participants were recruited at Stanford University School of Medicine, including 20 HC and 20 ASD. Children with ASD received a diagnosis based on scores from the Autism Diagnostic Interview-Revised (ADI-R) or the Autism Diagnostic Observation Schedule (ADOS) administered by a research reliable clinician. Both ASD and HC exclude participants with a history of any known genetic, psychiatric, or neurological disease.  Table 1 shows the details of the participants, including age, gender, FIQ, ADOS total, ADOS communication, and *p* value.

### 2.2. rs-fMRI Scan Parameters and Preprocessing

Participants were required to close their eyes but not sleep during data collection. All images were collected with TR = 2 s, TE = 30 ms, matrix size = 64 *∗* 64, flip angle of 80°, FOV = 20 cm, 29 slices, yielding 3.125 × 3.125 × 4.50 mm^3^ voxels for 180 time points, slice thickness = 3.5 mm, slice gap = 1.05 mm, field of view 240 mm, and resting-state scans consisting of *F* = 176 volumes.

The rs-fMRI raw data were preprocessed by using Python/FSL Resting State Pipeline platform [40]; then, the whole brain was divided into 90 brain regions by using Automated Anatomical Labeling (AAL) [41]. The preprocessing of the data mainly consists of the following seven steps: (1) data removal of the first 4 time points; (2) time layer correction; (3) head motion correction; (4) skull removal; (5) spatial standardization; (6) band-pass filtering; and (7) BOLD signal extraction for average time series of the brain region. After all these treatment, the BOLD time series of 90 brain regions at 176 sampling points were obtained.

### 2.3. Construction of Dynamic Functional Connectivity Brain Network

In order to observe the continuous change of BOLD signal with time in our work, we used sliding window technique [42, 43] to combine several BOLD values at adjacent sampling time. As a result, we got a small window which includes all the BOLD information of 90 brain regions in consecutive time as our state observation window for current sample time point. The window is moved from left to right according to time change in step size *l*. Here, the size of the sliding window *n* and the time step *l* can be determined by the attribution of experiment data. In this work, it is set to be *n* = 20 and *l* = 1. Then, the overall BOLD signal time series were divided into *g* = 157 (*g* = 176 − *n* + l) state observation windows. Now in each state observation windows, we got an *n* *∗* *N* matrix.

To explore the temporal synchronization and correlation of state observation windows, Pearson correlation analysis [32, 44] method is chosen to construct dynamic functional connectivity between brain regions in each sliding window. In order to make it conform to the network characteristics, threshold processing is performed in the process of constructing the network. Only when the correlation strength reaches a certain threshold, it is considered that there is a functional connection between the two brain regions. In order to select a reasonable threshold to construct a complete brain network functional connection, we use the small world of the network and the integrity of the brain function network as a method for determining the reasonable threshold range. Threshold estimation of all samples and selection of common intersections were used to determine reasonable thresholds. We take absolute values of all correlation coefficients and use the threshold th = 0.5 for network processing [45], which can be described by matrix *M* as follows:

(1) $$\begin{array}{c} M_{g}=\left[\begin{array}{cccc} M_{11} & M_{12} & \cdots & M_{u1}\\ M_{21} & M_{22} & \cdots & M_{u2}\\ \vdots & \vdots & \ddots & \vdots \\ M_{1u} & M_{2u} & \cdots & M_{uu} \end{array}\right]. \end{array}$$

If we take 90 brain regions as network nodes, here nodes = {1, 2, 3,…, 90}, *u* = 90, and take the correlation coefficients as the edge of the network, here edges = {*M*_11_, *M*_12_,…, *M*_*ij*_,…, *M*_*uu*_}, *u* = 90. A 90 × 90 state observation matrix *g* is obtained in each observation window. The state observation matrix is a diagonally symmetric matrix. The correlation coefficient between the brain regions can be regarded as the real-time feature of the brain network without considering the autocorrelation.

There are a total of 4005 components. A 4005-dimensional brain network state observation vector can be obtained by sequentially extracting the features in the diagonal correlation coefficient matrix from head to tail.

After completing all aforementioned steps, each resting state fMRI sample will get *g* vectors in the whole sampling interval; that is, we can get *g* × 4005 matrix *G*. The row vector of the matrix *G* represents the functional connection characteristics of the brain regions in a certain scanning time interval, and the matrix may reflect the dynamic process of the whole brain function network in entire data acquisition time.

### 2.4. Dimension Reduction of Brain Network State Observation Matrix Based on t-SNE

In the process of studying the dynamic characteristics of the brain network, the whole brain network state observation matrix is constructed. Because of the high dimensions up to 4005, it is very difficult for identification and analysis of the dynamic characteristics of brain network. In our work, t-distributed Stochastic Neighbor Embedding (t-SNE) [46] algorithm is used to reduce the dimensionality of the whole brain region network state observation matrix. The t-SNE algorithm is used in many studies and has achieved good dimensionality reduction and visualization [47, 48]. After using t-SNE, the high-dimensional whole brain region network state observation matrix is embedded into a two-dimensional space; as a result, the state distribution with time changing can be observed by clustering effect. Because the states change with time, the corresponding low-dimensional distribution looks like some continuous time points with intervals, and the last time point of each cluster indicates the state transition, which we called critical point of state transition in our research.

## 3. Methods

### 3.1. State Instantaneous Conversion Rate of Independent Samples

In this study, while using t-SNE algorithm to measure the difference between two probability distributions in high-dimensional space and low-dimensional space, the low-dimensional expression which embedded from high-dimensional brain network state observation matrix is obtained by the gradient descent method. Because the mapping result in two-dimensional space only shows the similarity degree of those states but not the position, the final cluster visualization results are random. In view of this randomness, we performed repetitious dimensionality reduction experiments on healthy samples and counted the number of state groups and the critical point of state transition.

We set *m* ∈ *N*^+^ as the number of different states and *S*_*p*_ as the corresponding states, *p*={1,2,3,…, *m*}, and then in each clustering experiment an independent sample data can obtain *m* − 1 state transition critical points *D*_*q*_, *q* = {1, 2, 3,…, *m* − 1}. The high-dimensional brain network state observation matrix is subjected to dimensionality reduction clustering experiment for 1000 times. The time point *k* (0 < *k* ≤ *g*) at each state of each subject *S*_*p*_ to *S*_*p*+1_ is recorded as *D*_*q*_, and then we can get the *D*_*q*_ set *G*_*q*_ of all 1000 times experiments. By counting the frequency *N*_*k*_ of the state transition critical point *k* element in each *G*_*q*_ set corresponding to each *D*_*q*_ and observing the time point distribution position of all *D*_*q*_ elements in *G*_*q*_ set, each brain network state transition time interval *P*_*m*−1_=(*t*_1_, *t*_2_) can be estimated.

Based on the aforementioned statistics for both the HC and ASD groups, we calculated the average interval coincidence degree *Q* in each sample and compared the arithmetic mean of the coincidence values of all the samples in the HC group and ASD group:

(2) $$\begin{array}{c} \bar{Q}=\frac{1}{n}{\overset{n}{\underset{y=1}{\sum }}\left(\frac{\sum_{x=1}^{m-2}R_{x}}{g}\right)}_{y}, \end{array}$$

where *R* is the overlap value of the adjacent state transition interval, *g* is the number of observation time points, and *n* is the number of samples in the group.

Based on the above equation, we establish the state transition trend of the state instantaneous conversion rate in the time interval as follows:

(3) $$\begin{array}{c} \lambda_{k}=\frac{\sum_{k=0}^{t_{1}}N_{k}}{\sum_{k=t_{1}}^{t_{2}}N_{k}*\left(j-i\right)}, \end{array}$$

where *N*_*k*_ is the frequency at which the brain network state transition critical time point *k* element appears in the *G*_*q*_ set corresponding to each *D*_*q*_.

### 3.2. Neighbor Topology Overlap Coefficient between States

Through the above research, we have found that the state transition of brain network shows a certain regularity in time. In the existing research, some studies divide the state of brain network differently, but we believe that the division of brain network state will produce difference due to individual differences. At the time of conversion, it is impossible to explore the regularity of brain network state transition. We propose that the state transition of the brain network is not instantaneous but in an interval. From this, we divide the entire time period into *s* states, including *m* steady state intervals and *s*-*m* state transition intervals. The entire observation interval *g* is divided into *s* state intervals of *S*_1_, *S*_2_, *S*_3_,…, *S*_*s*_. *S*_2*f*−1_ is the state steady interval, and *S*_2*f*_ is the state transition interval (*f* ∈ *N*^+^). The corresponding positions of the matrices in each state are added and averaged as $\bar{S_{s}}$ matrix:

(4) $$\begin{array}{c} \bar{S_{s}}=\left[\begin{array}{cccc} \bar{\sum M_{11}} & \bar{\sum M_{12}} & \cdots & \bar{\sum M_{1u}}\\ \bar{\sum M_{21}} & \bar{\sum M_{22}} & \cdots & \bar{\sum M_{2u}}\\ \vdots & \vdots & \ddots & \vdots \\ \bar{\sum M_{u1}} & \bar{\sum M_{u2}} & \cdots & \bar{\sum M_{uu}} \end{array}\right]. \end{array}$$

*n* = 20 samples are summed and averaged as brain network state matrix $\bar{Z_{s}}$:

(5) $$\begin{array}{c} \bar{Z_{s}}=\left[\begin{array}{cccc} \frac{\sum_{1}^{n}{\left(\bar{\sum M_{11}}\right)}_{n}}{n} & \frac{\sum_{1}^{n}{\left(\bar{\sum M_{12}}\right)}_{n}}{n} & \cdots & \frac{\sum_{1}^{n}{\left(\bar{\sum M_{u1}}\right)}_{n}}{n}\\ \frac{\sum_{1}^{n}{\left(\bar{\sum M_{21}}\right)}_{n}}{n} & \frac{\sum_{1}^{n}{\left(\bar{\sum M_{22}}\right)}_{n}}{n} & \cdots & \frac{\sum_{1}^{n}{\left(\bar{\sum M_{u2}}\right)}_{n}}{n}\\ \vdots & \vdots & \ddots & \vdots \\ \frac{\sum_{1}^{n}{\left(\bar{\sum M_{u1}}\right)}_{n}}{n} & \frac{\sum_{1}^{n}{\left(\bar{\sum M_{u2}}\right)}_{n}}{n} & \cdots & \frac{\sum_{1}^{n}{\left(\bar{\sum M_{uu}}\right)}_{n}}{n} \end{array}\right]=\left[\begin{array}{cccc} a_{11} & a_{12} & \cdots & a_{1u}\\ a_{21} & a_{22} & \cdots & a_{2u}\\ \vdots & \vdots & a_{ij} & \vdots \\ a_{u1} & a_{u2} & \cdots & a_{uu} \end{array}\right]. \end{array}$$

The neighbor network topology overlap coefficient is mainly used to represent the similarity of two network topologies in a time series network [49]. This paper uses the neighbor network topology overlap coefficient to interpret the network topology changes with state over time. The topology overlap coefficient of each state is calculated by using the neighbor network topology overlap coefficient. Network topology matrices of *s∗s* are obtained. The topology overlap coefficient is as follows:

(6) $$\begin{array}{c} C_{i}^{\left(Z_{s},Z_{s-1}\right)}=\left\{\frac{\sum_{j}\left(1-\left|a_{ij}\left(\bar{Z_{s}}\right)-a_{ij}\left(\bar{Z_{s-1}}\right)\right|\right)a_{ij}\left(\bar{Z_{s}}\right)a_{ij}\left(\bar{Z_{s-1}}\right)}{\sqrt{\left[\sum_{j}a_{ij}\left(\bar{Z_{s}}\right)\right]\left[\sum_{j}a_{ij}\left(\bar{Z_{s-1}}\right)\right]}}\right., \end{array}$$

(7) $$\begin{array}{c} C^{\left(Z_{s},Z_{s-1}\right)}=\frac{1}{u}\overset{u}{\underset{i=1}{\sum }}C_{i}^{\left(Z_{s},Z_{s-1}\right)}. \end{array}$$

**Figure 1:**
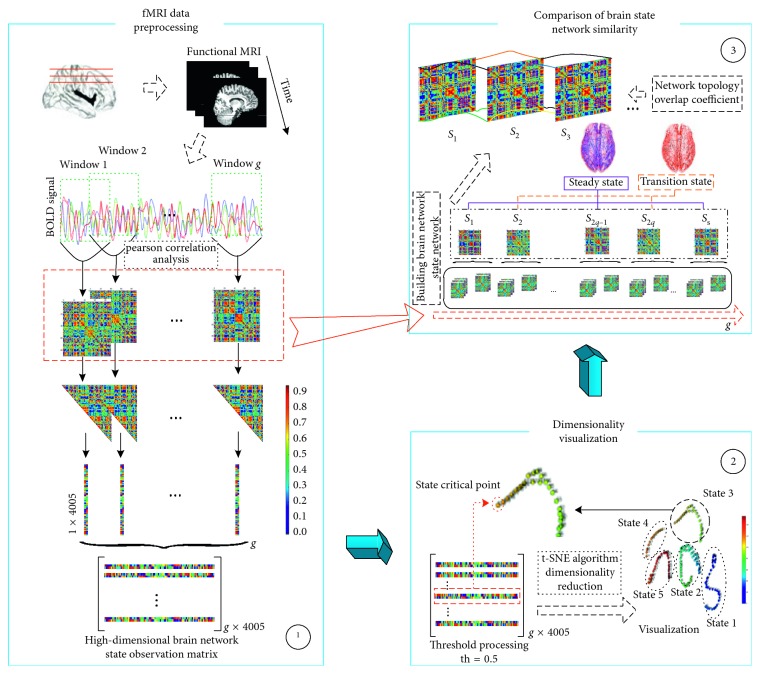
Workflow. ① Preprocessing of fMRI data and construction of high-dimensional brain network state observation matrix. ② Dimensionality reduction of high-dimensional brain network state observation matrix and analysis of state transition critical point. ③ Constructing brain state network and using network topological coefficients to study the event characteristics of brain state.

Formula (6) calculates the topological similarity of node *i* in state networks $\bar{Z_{s}}$ and $\bar{Z_{s-1}}$, (0 < *s* ≤ 9, *s* ∈ *N*^+^; 0 < *i* ≤ 90, *i* ∈ *N*^+^). Formula (7) calculates the average similarity of the topological similarity of all nodes in the entire network and measures the similarity between the network $\bar{Z_{s}}$ and the network $\bar{Z_{s-1}}$. The value of *C*^(*Z*_*s*_,  *Z*_*s*−1_)^ is in the range [0, 1], and the more the *C*^(*Z*_*s*_,  *Z*_*s*−1_)^ value is, the more similar the state networks $\bar{Z_{s}}$ and $\bar{Z_{s-1}}$ are. A 9*∗*9 neighbor network topology overlap matrix is obtained and visually represented. The state topology overlap coefficient matrices of the HC group and the ASD group are analyzed separately, and their differences are compared (Figure 1).

## 4. Experimental Result and Discussion

### 4.1. Brain Network State Transition Visualization Based on t-SNE

**Figure 2:**
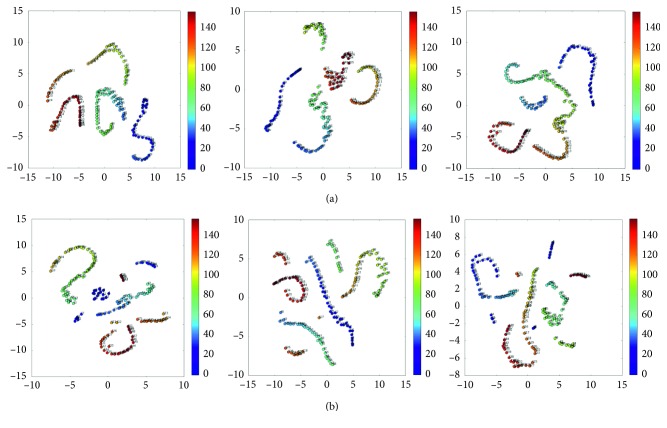
Reduced-dimensional visualization of 3 samples from the (a) HC group and (b) ASD group.

With the method presented in Section 2.4, T-SNE algorithm was used in dimensionality reduction and visualization for the brain network state observation matrix data of different subjects in the HC group and ASD group, respectively (Figure 2). The clustering results in both groups showed chain distribution expression with time characteristics, and from these results, we can find some differences between the HC group and ASD group. The clustering results of subjects in the HC group showed some common features: the data points in adjacent time are distributed closely, the critical points are almost in the same time frame, and the elements in the same states sets are very similar. While the clustering results of subjects in the ASD group are not universal enough, there are many scatter time points and the state clustering results has no obvious regularity.

According to the results of the dimension reduction experiments of the HC group, although the clustering results of different individual samples are in different spatial positions because of the randomness, the similarity can be observed in the cluster number and the state transition critical points, which reflected the brain network state transition regularity with time. Although the ASD group and the HC group have significant differences in dimensionality reduction results, we also found that the ASD group has been divided into different groups by the clustering results. We think that there is also state transition in the ASD group, but there is no common law.

### 4.2. State Instantaneous Conversion Rate

**Figure 3:**
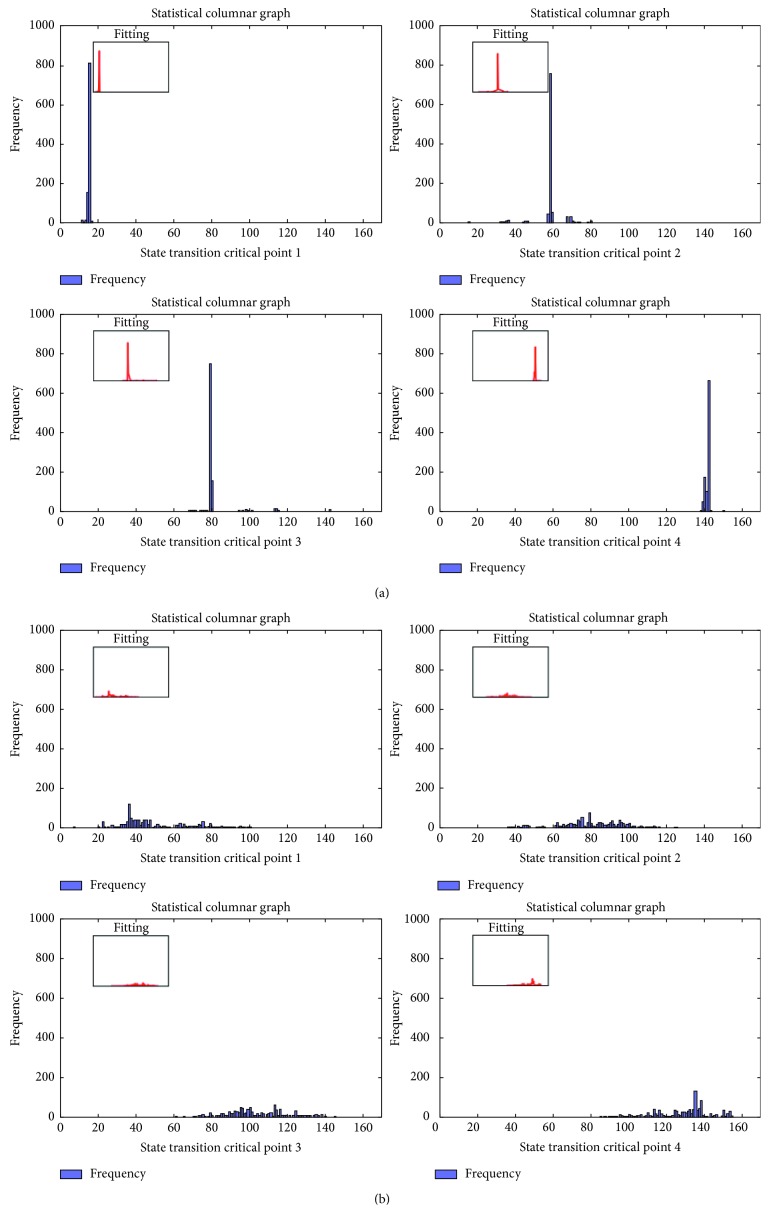
Statistical frequency map of sample 1 brain network state critical point in the (a) HC group and (b) ASD group.

In order to study the regularity of state transition, the critical points are analyzed based on Section 4.1. With the number of state groups *m* = 5, embedding and clustering experiments are performed for 1000 times on each independent data samples of the HC group and ASD group, and the state changing of *S*_*p*_ to *S*_*p*+1_ in the experiment is observed. The time point *k* (0 < *k* < *t*) at which the transition critical point *D*_*q*_ is located and counted as *G*_*q*_ (0 < *m* ≤ 5) set. The frequency *N*_*k*_ of each state transition critical time point *k* in the *G*_*q*_ set is shown in Figure 3. In the HC group, value of *N*_*k*_ shows obviously difference for different state transition critical points, especially the maximum value is far more than the other values in the *G*_*q*_ set, and there are only few states near this state transition critical time point *k* corresponding to the maximum value. At the interval between those maximum values, the *N*_*k*_ is very small, so each state transition period can be observed independently. While in the ASD group, the difference between state transition critical point frequency values is small, there are no clear signs for the occurring of state transition. The time interval is wide ranging, and there is an overlapping portion between neighboring states.

According to the statistical results of the HC group, the state transition critical points are concentrated at certain time points and their surroundings. The state transition intervals are formed naturally. Each transition interval is independent of other intervals, and only a few overlaps occur. From these regular patterns, we conclude that for HC sample, each brain network state is independent and the state transition is regular and stable. To the contrary, although the state transition can be exhibited in above sample experiments in the ASD group, the statistical results display that the range of the state transition critical point *k* is too wide to distinguish, and as a result too much overlap appeared between adjacent states. This kind of overlap represents instability of the brain network during the status transformation; at the meantime, the results of different subjects in the ASD group did not show common regularities.

**Figure 4:**
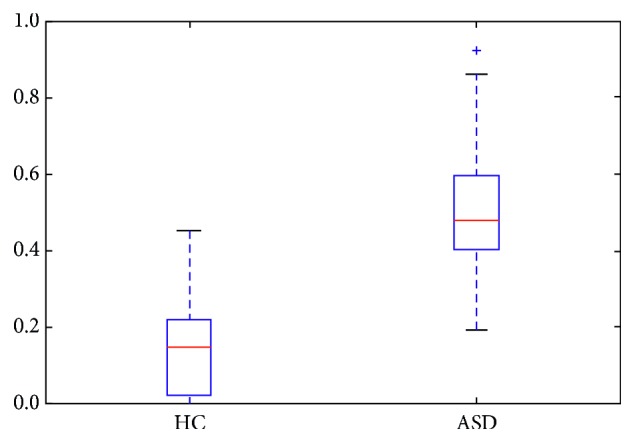
Comparison of coincidence coefficient of state transition interval between the HC group and ASD group. Analysis of the significant differences between the two groups using analysis of variance (ANOVA) is shown in Table 2.

In order to verify that the above samples are not specific, the length of the state transition interval was counted and the average state transition interval coincidence degree was calculated for all samples of both the HC group and ASD group, as shown in Figure 4. The interval overlap of brain network in the ASD group was significantly higher than that in the HC group excluding the sample specificity. At the meantime, analysis of variance (ANOVA) was used to test the difference in data (*F* = 54.836, *p* value ≈ 0), and the results are shown in Table 2.

**Figure 5:**
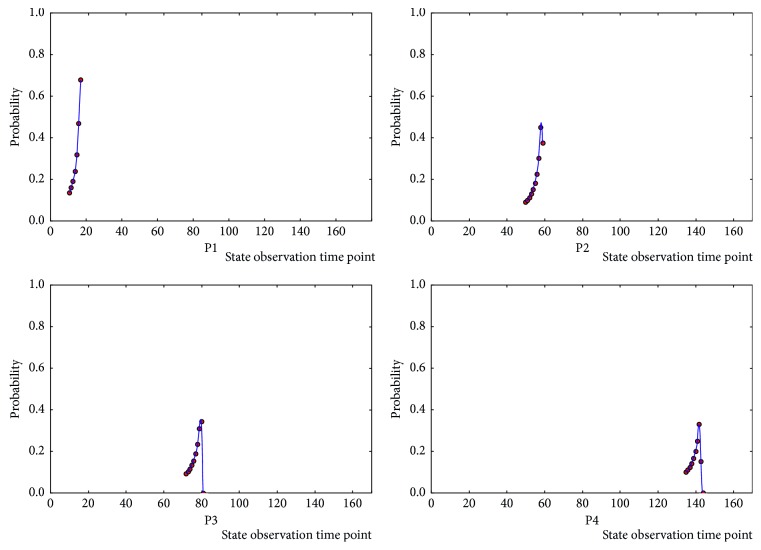
Instantaneous conversion rate of sample 1 in the HC group.

The existing research studies on brain network state regard the state transition was finished in a specific time point, which means the transition is instantaneous. While in the above experiments, by counting the state transition critical point, we noticed that the state transition critical point *k* from *S*_*p*_ to *S*_*p*+1_ state transition forms regional range *P*_*q*_ (*t*_1_, *t*_2_). In this paper, we propose that the state transition appeared in a time interval rather than in a certain time. Here, we construct a brain network state instantaneous conversion rate to observe the state transition trend of the HC group in the interval *P*_*q*_ (*t*_1_, *t*_2_) (Figure 5). The results show that the HC group samples are not instantaneously converted but gradually converted in the *P*_*q*_ (*t*_1_, *t*_2_) interval. Therefore, we recognize that the brain network state has a state transition interval during the conversion process, which is also one of the brain network features and cannot be ignored.

### 4.3. Neighbor Topology Overlap Coefficient between States

**Figure 6:**
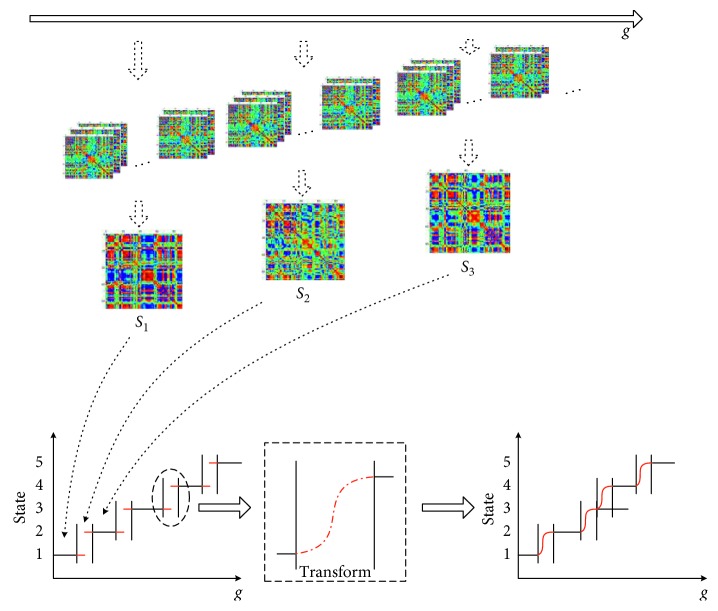
Schematic diagram of brain network state construction and gradual transition in state interval.

In order to continue to explore the time-varying characteristics of the brain network state, based on the above analysis, we constructed the entire observation process with *s* = 9 which includes *m* = 5 steady state periods and *s* − *m* = 4 state transition intervals in our experiments. These 9 intervals can be shown as follows: *S*_1_, *S*_2_, *S*_3_,…, *S*_9_. *S*_1_ = {*M*_1_, *M*_2_,…, *M*_10_}; *S*_2_ = {*M*_10_, *M*_11_,…, *M*_15_}; *S*_3_ = {*M*_16_, *M*_17_,…, *M*_50_}; *S*_4_ = {*M*_51_, *M*_52_,…, *M*_55_}; *S*_5_ = {*M*_56_, *M*_57_,…, *M*_85_}; *S*_6_ = {*M*_86_, *M*_87_,…, *M*_90_}; *S*_7_ = {*M*_91_, *M*_92_,…, *M*_125_}; *S*_8_ = {*M*_126_, *M*_127_,…, *M*_130_}; and *S*_9_ = {*M*_131_, *M*_132_,…, *M*_157_}. By reconstructing the brain state network from the group analysis of the dynamic brain function network and exploring the structural topological similarity between different brain functional network states, the network topology overlap coefficient can be calculated to express the brain network state characteristics over time in network topology. In our research, this index was used to study the differences between HC and ASD in dynamic brain function network state so that to verify the effectiveness of the proposed method (Figure 6).

**Figure 7:**
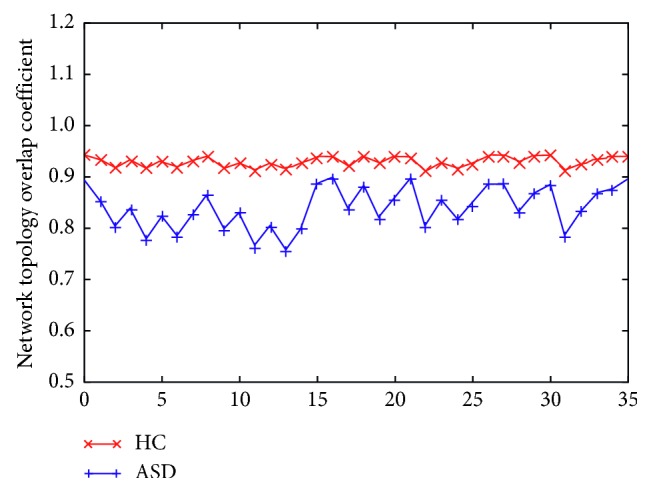
Comparison of brain state network topological coefficients between the HC group and ASD group. Analysis of the significant differences between the two groups using analysis of variance (ANOVA) is shown in Table 3.

**Figure 8:**
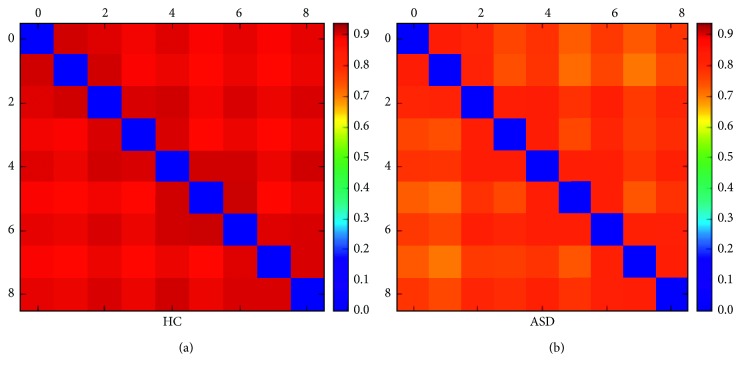
Visualization of topological overlap coefficient of brain network state network in the (a) HC group and (b) ASD group.

The time-varying characteristics of the brain network state of the HC group and ASD group showed significant differences. Overall, the degree of network topology overlap of the HC group is much higher than that of the ASD group in any two kinds of states, as shown in Figure 7. Analysis of variance (ANOVA) was used to test the difference in network topology overlap coefficient of the HC group and ASD group (Table 3). The network topology overlap coefficient between the *s* = 9 states in the HC group shows a clear common regularity (Figure 8(a)).

The similarity of the network topology in all stable intervals is more remarkable than the similarity between the stable interval and the transition interval, and it is also higher than the network topology similarity between the transition intervals. The similarity between the network topology of the stable interval and the transition interval is higher than the network topology similarity in all the transition intervals (Table 4).

The network topology overlap coefficient of these nine states of the ASD group indicates that the similarity of the network topology in all stable intervals is higher than the network topology similarity between the stable interval and the transition interval, and it is also higher than the similarity of the network topology in all the transition intervals. However, the similarity of the network topology in the stable intervals of the ASD group diminishes gradually over time. The network topology between all the transition interval is the weakest, and the similarity decreases with time (Table 5).

In the comparative analysis of the experimental results, the network similarity between the stable and transition intervals of the HC group showed some difference, but the similarity between all stable intervals and all transition intervals did not show any obvious discriminate. On the other hand, the ASD group has time-variation in network topology similarity, and the overall network similarity is weaker than that of the HC group (Figure 8(b)).

## 5. Conclusions

In this work, the time-varying characteristics of dynamic brain function networks are studied, and a dynamic brain network state observation matrix is constructed. The dimensionality reduction and visualization of this high-dimensional matrix is processed; then the different states can be grouped automatically based on the clustering result, and the state transition critical points can be counted. According to the statistical results, the instantaneous conversion rate is established to observe the transition trend of the state within intervals.

Here, we propose a hypothesis that the state transition is completed in a range but not instantaneous. To testify this viewpoint, we construct the instantaneous hopping rate to observe the state transition trend between intervals. We think that the processes of state transition are also regarded as states existing in the activities of the brain network and cannot be ignored. By constructing a state network including both steady states and transition states, the network topology overlap coefficient is used to represent the network topology similarity between states. The network topology similarities between different states are computed to study the time-varying characteristics of brain networks. The difference between HC and ASD in brain network dynamic features was analyzed by the method in this paper, and the effectiveness of the method was verified.

## Figures and Tables

**Table 1:** Demographics.

	ASD (*n* = 20)	HC (*n* = 20)	*p* value
Age (years) (range)	9.96 ± 1.58 (7.5–12.9)	9.95 ± 1.60 (7.8–12.4)	0.959
Gender (M/F)	16/4	16/4	—
FIQ	112.55 ± 17.79	112.10 ± 15.37	0.531
ADOS total (range)	11.73 ± 3.62 (7–18)	—	—
ADOS communication (range)	3.57 ± 1.53 (2–7)	—	—

**Table 2:** Variance analysis result of interval overlap coefficient.

	Sum of squares	Degrees of freedom	Mean of squares	*F*	Sig.
Between groups	1.448	1	1.448	54.836	0.000
Intragroup	1.003	38	0.026	—	—
Total	2.451	39	—	—	—

**Table 3:** Variance analysis result of two groups of network topology overlap coefficients.

	Sum of squares	Degrees of freedom	Mean of squares	*F*	Sig.
Between groups	0.145	1	0.145	158.154	0.000
Intragroup	0.064	70	0.001	—	—
Total	0.209	71	—	—	—

**Table 4:** Topological overlap coefficient between brain network states of the HC group.

States	Steady states					Transition states			
	*S*_1_	*S*_3_	*S*_5_	*S*_7_	*S*_9_	*S*_2_	*S*_4_	*S*_6_	*S*_8_
*S*_1_	0.000	0.933	0.930	0.929	0.929	0.941	0.918	0.915	0.918
*S*_2_	0.941	0.938	0.925	0.924	0.925	0.000	0.915	0.911	0.913
*S*_3_	0.933	0.000	0.938	0.937	0.937	0.938	0.935	0.920	0.925
*S*_4_	0.918	0.935	0.936	0.925	0.924	0.915	0.000	0.910	0.913
*S*_5_	0.930	0.938	0.000	0.940	0.938	0.925	0.936	0.938	0.926
*S*_6_	0.915	0.920	0.938	0.942	0.922	0.911	0.910	0.000	0.911
*S*_7_	0.929	0.937	0.940	0.000	0.937	0.924	0.925	0.942	0.931
*S*_8_	0.918	0.925	0.926	0.931	0.937	0.913	0.913	0.911	0.000
*S*_9_	0.929	0.937	0.938	0.937	0.000	0.925	0.924	0.922	0.937

**Table 5:** Topological overlap coefficient between brain network states of the ASD group.

States	Steady states					Transition states			
	*S*_1_	*S*_3_	*S*_5_	*S*_7_	*S*_9_	*S*_2_	*S*_4_	*S*_6_	*S*_8_
*S*_1_	0.000	0.852	0.836	0.821	0.827	0.897	0.801	0.775	0.781
*S*_2_	0.897	0.863	0.828	0.802	0.797	0.000	0.793	0.760	0.753
*S*_3_	0.852	0.000	0.896	0.880	0.855	0.863	0.884	0.835	0.817
*S*_4_	0.801	0.884	0.896	0.854	0.841	0.793	0.000	0.800	0.815
*S*_5_	0.836	0.896	0.000	0.884	0.867	0.828	0.896	0.886	0.830
*S*_6_	0.775	0.835	0.886	0.883	0.832	0.760	0.800	0.000	0.781
*S*_7_	0.821	0.880	0.884	0.000	0.872	0.802	0.854	0.883	0.865
*S*_8_	0.781	0.817	0.830	0.865	0.893	0.753	0.815	0.781	0.000
*S*_9_	0.827	0.855	0.867	0.872	0.000	0.797	0.841	0.832	0.893

## Data Availability

The data used in this study are obtained from the ABIDE database: http://fcon_1000.projects.nitrc.org/indi/abide/.
